# Mathematical Modelling to Predict Oxidative Behaviour of Conjugated Linoleic Acid in the Food Processing Industry

**DOI:** 10.3390/foods2020274

**Published:** 2013-06-20

**Authors:** Aitziber Ojanguren, Josune Ayo

**Affiliations:** Food Research Division, AZTI-Tecnalia, Technology Park of Biscay, Astondo Bidea, Building 609, 48160 Derio, Biscay, Spain; E-Mail: aitziber.ojanguren@gmail.com

**Keywords:** three-level full factorial design, bioactive conjugated isomers, frying, oil stability index (OSI)

## Abstract

Industrial processes that apply high temperatures in the presence of oxygen may compromise the stability of conjugated linoleic acid (CLA) bioactive isomers. Statistical techniques are used in this study to model and predict, on a laboratory scale, the oxidative behaviour of oil with high CLA content, controlling the limiting factors of food processing. This modelling aims to estimate the impact of an industrial frying process (140 °C, 7 L/h air) on the oxidation of CLA oil for use as frying oil instead of sunflower oil. A factorial design was constructed within a temperature (80–200 °C) and air flow (7–20 L/h) range. Oil stability index (Rancimat method) was used as a measure of oxidation. Three-level full factorial design was used to obtain a quadratic model for CLA oil, enabling the oxidative behaviour to be predicted under predetermined process conditions (temperature and air flow). It is deduced that temperatures applied in food processes affect the oxidation of CLA to a greater extent than air flow. As a result, it is estimated that the oxidative stability of CLA oil is less resistant to industrial frying than sunflower oil. In conclusion, thanks to the mathematical model, a good choice of the appropriate industrial food process can be selected to avoid the oxidation of the bioactive isomers of CLA, ensuring its functionality in novel applications.

## 1. Introduction

Conjugated linoleic acid (CLA) is a term used to denote a group of isomers of linoleic acid (*cis*-9, *cis*-12-octadecadienoic acid). The characteristic that binds these isomers together is that, unlike linoleic acid, the two double bonds along the 18-carbon fatty acid chain are not separated by a methylene carbon and hence, are termed “conjugated”. Scientific interest in CLA has risen exponentially since its discovery [1] in the 1980’s, mainly due to its beneficial effects in weight management. There is increasing evidence that dietary intake of 3.4 g of bioactive CLA isomers per day, may decrease body fat and increase lean body mass [2].

At the same time, there is a growing interest by industry in innovating and marketing products that may provide health benefits beyond basic nutrition. Consequently, design of new health products based on CLA is a good opportunity to gain market share.

Operational parameters of several steps in industrial processes typically involve using high temperatures in contact with air. It is well known that high temperatures and oxygen concentration present in air are the most important factors affecting unsaturated fatty acid degradation. Consequently, CLA with two double bonds may undergo oxidation during processing, which results in the loss of its beneficial effects. Therefore, the oxidative stability of CLA should be considered before choosing the appropriate industrial food process.

Several studies in the literature have been reported describing the influence of oxygen and temperature on oxidative stability of different oils. So far, oxidation pathways of CLA are unclear and the mechanisms involved remain controversial [3,4,5]. It was reported that CLA was extremely unstable when heated in air presence [6] decomposing to furan fatty acids [7]. Some authors [3,5] have found significant levels of volatile compounds in CLA oxidized oil and Suzuki *et al.* [8] observed dimer and polymer formation in the oxidation of CLA. Recently, Giua *et al.* [9] have observed geometrical isomerization of *cis*,*trans* and *trans*,*cis* CLA to *trans*,*trans* isomers. This controversy may perhaps be partly explained by the lack of studies performed, therefore more investigation is needed into aspects concerning CLA oxidation related to industrial processing factors (temperature and oxygen).

In this sense, statistical and mathematical techniques for designing experiments are useful tools to estimate the industrial behavior of oils. In particular, three-level full factorial design has been proposed to determine the influences of temperature and air and their interactive influences on the stability of oil with high CLA content. The aim of this study is to build a model and predict, on a laboratory scale, the industrial oxidative behavior of oil with high CLA content, by controlling the limiting factors of food processing with mathematical tools. The statistical model aims to estimate the impact of an industrial frying process on the oxidation of CLA oil as a substitute to sunflower oil.

## 2. Experimental Section

### 2.1. Materials

High content CLA oil (Clarinol^®^ G-80) was supplied by Lipid Nutrition (Stepan Lipid Nutrition; Koog aan de Zaan, Netherlands). Clarinol^®^ G-80 contained a 78.8% ± 0.2% of the bioactive isomers (c9,t11 and t10,c12) of CLA, as triglyceride form in a 50:50 ratio. Other fatty acids present in CLA oil were palmitic (4.8% ± 0.2%), stearic (3.1% ± 0.1%), oleic (10.4% ± 0.2%), linoleic (1.3% ± 0.1%) and other conjugated linoleic fatty acids (1.6% ± 0.1%). The main unsaturated fatty acids present in the sunflower oil used in this study were linoleic acid (59.5% ± 0.1%) and oleic acid (28.5% ± 0.2%), followed by palmitic (4.5% ± 0.1%) and stearic acid (7.5% ± 0.1%).

### 2.2. Determination of Oil Stability Index (OSI)

The oil stability index (OSI) was carried out with the model 743 Rancimat equipment (Metrohm, Herisau, Switzerland). The OSI was obtained by subjecting oils (2.5 ± 0.1 g) to the operational parameters of temperature and oxygen measured as the air flow applied on the oils in Rancimat tubes. The volatile oxidation products were stripped from the oil and dissolved in cold water, increasing its conductivity. The time taken until there is a sharp increase of conductivity is OSI and it was determined by the intersection of the baseline with the tangent to the conductivity curve [10]. 

In order to estimate the impact of an industrial frying process on CLA oil, the oxidative stability index of CLA and sunflower oils were also compared in triplicate at 140 °C and 7 L/h.

### 2.3. Experimental Design

Three-level full factorial design was used to gain maximal information about the influence of temperature and oxygen on the oxidation stability of CLA oil. The design was employed to investigate the oxidation behaviour of CLA oil measured as the variation of the OSI with respect to operating parameters temperature (T) and air flow (AF). Thirteen experimental settings consisting of nine points and four central points were generated with two factors and three levels by using Statgraphics plus 5.0 (StatPoint Technologies, Inc.; Warrenton, VA, USA). The variables and their concentration ranges were: temperature from 80 °C to 200 °C and oxygen content as AF from 7 to 20 L/h.

In order to normalize data distribution and obtain a better fit of the model, the logarithm transformation was applied to the response (log(OSI)). The experiments were performed under conditions established by the three level factorial design matrix (Table 1). Experiments were carried out in a random order in triplicate.

**Table 1:** Three level factorial design for the induction period of the conjugated linoleic acid (CLA) oil’s stability index, with corresponding observed and predicted values.

Run *n*°	Variable codes		Process variables		Log(OSI) for CLA	
	X_1_	X_2_	Temperature (°C)	AF (L/h)	Observed	Predicted
1	1	1	200	20	0.64 ± 0.03	0.69
2	0	−1	140	7	1.50 ± 0.03	1.49
3	0	1	140	20	1.47 ± 0.03	1.37
4	0	0	140	13	1.45 ± 0.03	1.43
5	1	−1	200	7	1.01 ± 0.02	1.01
6	0	0	140	13	1.37 ± 0.06	1.43
7	0	0	140	13	1.37 ± 0.05	1.43
8	−1	−1	80	7	3.49 ± 0.01	3.47
9	−1	0	80	13	3.52 ± 0.01	3.51
10	1	0	200	13	0.89 ± 0.03	0.85
11	−1	1	80	20	3.53 ± 0.01	3.55
12	0	0	140	13	1.40 ± 0.01	1.43
13	0	0	140	13	1.46 ± 0.04	1.43

AF = Air Flow.

The analysis of *p*-values, for linear, quadratic and interaction effects of temperature and air flow on OSI were analyzed. The significance of each parameter and the interaction strength was checked by analyzing the *p*-values. 

## 3. Results and Discussion

Among the factors studied, the linear and quadratic terms of temperature were the most significant (*p* < 0.0001) effects on the transformed response. The interaction term between temperature and air flow (T × AF) was also significant (*p* < 0.01) to the model. The linear term of AF was the least significant (*p* = 0.031) factor. On the other hand, the quadratic air flow (AF^2^) term coefficient had no significant influence (*p* = 0.495) on log(OSI), so it was omitted from the final model. All significant terms were included in the equation.

The mathematical expression obtained for the transformed oil stability index of the CLA oil as a function of temperature and air flow was expressed by the following equation:

log(OSI) = 1.43108 − 1.33238 × T − 0.0592154 × AF + 0.748602 × T^2^ − 0.10176 × T × AF

The model was checked using a numerical method employing the coefficient of determination (*R*^2^) and adjusted *R*^2^ (*R*^2^adj). The *R*^2^ value (0.998), found to be close to 1.0, denotes a high correlation between the observed and predicted values. Furthermore, the *R*^2^adj of the model (0.997) was also close to the *R*^2^ values. Thus, the polynomial model given in equation provides an excellent explanation of the relationship between the independent variables (T and AF) and the transformed response (log(OSI)). The lack of fit test was not significant (*p* = 0.250), which denotes the reliability of the model. Hence the model accurately represents the effect of temperature and air flow on the oxidation of the CLA oil (log(OSI)) using statistical optimization.

**Figure 1:**
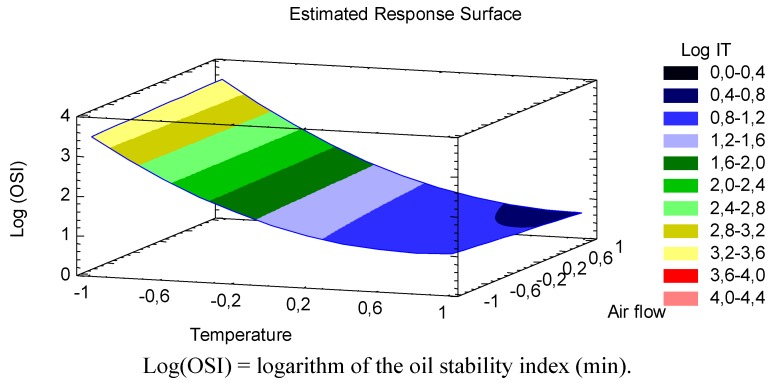
Curve showing predicted response surface of log oil stability index of CLA as a function of temperature (°C) and air flow (L/h).

The best way to visualize the influence of the factors on the transformed response is to draw surface response plots of the model. The three-dimensional response surface showing predicted response of log(OSI) of CLA oil as a function of temperature and air flow is given in Figure 1.

In line with the *p*-values of the terms in the mathematical model, Figure 1 showed that the logarithm of CLA oil stability index significantly decreased with increasing temperature. Moreover, the effect of the quadratic term of temperature was represented as a slight curvature in the transformed response, showing a tendency of the stability index of the CLA oil to stay constant at especially high temperatures (>170 °C). Consequently, the habitual temperatures achieve in industrial food treatments adversely affects the oxidative stability of this functional oil, in particular between 80 °C and 150 °C where a steeper negative slope was depicted. Giua *et al.* [9] have recently demonstrated that among the volatile oxidation products derived from CLA oxidation, saturated aldehydes were generally the mainly represented ones. Actually, some authors have reported that heptanal and hexanal were the major volatile oxidation product derived from CLA oxidation, with an additional relevant presence of *trans*-2-nonena [3,5]. These volatile compounds formed during accelerated oxidation are trapped during measurement by the Rancimat assay, increasing the distilled water conductivity. In this respect, Giua *et al.* [9] also observed oxidative reactions during a heat treatment process at 180 °C. They showed an initial increase in the formation of aldehydes (the most represented volatile compounds) after 15 min of heating treatment. According to our equation, the sharp increase of total volatile products derived from CLA oil oxidation (at 180 °C and an air flow of 7 L/h) is performed earlier, in particular after 9.95 min. This time variation would probably due to a different degree of oxygen exposure between two studies. Indeed, our study showed more unfavorable air flow conditions. In this regard, the effect of air flow on CLA oil’s stability was also discussed. 

According to the *p* values obtained in this study, although the liner term of the air flow was statistically significant, the impact on the transformed (log) CLA oil stability index was less relevant than temperature. In fact, it was a barely visible effect in Figure 1, in view of a slight linear decrease of log(OSI) as increasing the air flow, and the presence of oxygen, especially at high temperatures. As deduced from the lack of significance (*p* ≥ 0.05) of AF^2^ term to the model, no quadratic effect of the air flow was observed and therefore, for this factor, there was not a curvature in the response surface (Figure 1) where a linear model was performed. Choe and Min [11] showed that the stability index of unsaturated fatty acids is less oxygen-dependent when they are exposed to an excess amount of air as in the Rancimat equipment. So, it is likely the effect of oxygen (AF) on oxidation (log(OSI)) was of minimal significance (*p* = 0.031) in case of linear term or no significance (*p* ≥ 0.05) regarding quadratic term (AF^2^), due to air saturation. However, the influence of interactions between temperature and air flow on the model was more significant than air flow alone. As shown in Figure 1, changes in response due to an increase in air flow are different when increasing the temperature, *i.e.*, at low temperatures the oxidation behavior is independent of air flow variation, meanwhile at high temperature an increase in air flow have an negative effect on the transformed stability index of CLA oil, reducing to a large extent, the oxidation time. It has been pointed out that CLA is extremely susceptible to autoxidation when it was exposed to air and temperature. Yang *et al.* [6] showed that CLA added canola oil (containing 10% of CLA) was rapidly oxidized and reduced to 14% after 35 h heating at 90 °C. On the other hand, Chen *et al.* [12] showed that CLA was completely degraded after heating at 150 °C and 200 °C for 40 and 20 min, respectively.

Thus, considering the main factors (temperature and presence of oxygen) involved in food processing, this mathematical modeling aims to estimate practical data to predict, on a laboratory scale, the industrial oxidative behavior in industrial heat treatment, in particular frying, of oil with high CLA content. Controversial conclusion had been reported about the use of the Rancimat test for predicting the oxidative stability of oils under various heating processes like industrial frying. Some authors [13] have shown that the results obtained by this method are not directly transposable to real frying conditions. However, under the Rancimat test conditions, relative oxidative stability index and the kinetic parameters of the oxidation of some vegetable oils had been successfully compared under various heat (100–130 °C) processing conditions [14]. Consequently, in this study the mathematical model has been used as a tool to compare the oil stability of a functional oil rich in CLA with typically used sunflower oil during simulated frying conditions. 

Using the mathematical model, predicted stability index of CLA oil under frying conditions (140 °C and 7 L/h of air flow) will be reached after 30.63 min, a deviation of a 3.06% from the observed value (31.60 ± 2.43). This data led to assume that the model provide a very appropriate reproducibility of the prediction of CLA oil’s oxidative stability index, under the conditions of the Rancimat test that simulate deep-frying process.

On the other hand, observed stability index of sunflower oil (45.60 ± 2.08 min) was significantly higher than that observed for CLA oil under the same conditions. This means that CLA oil is almost 31% less stable than sunflower oil under these operational parameters. The difference in stability index observed seem to arise from the greater content of polyunsaturated fatty acids in the CLA oil (80.1%) compared to the sunflower oil (59.5%). Moreover, the presence of conjugated double bonds in CLA oil may have also influenced on its stability. In fact, it has been reported that CLA with two conjugated double bonds was more susceptible to oxidation than oleic [12] and also to linoleic acid [4] (two non-conjugated double bonds), when exposed to temperature and air. In this sense, it has been reported that the oxidation mechanism for the substrates with conjugated double bonds was different from that of linoleate and oleate [15]. Due to resonance delocalization*,* CLA can readily donate an electron or hydrogen to form a free radical intermediate of CLA [6]. In fact, the rate of the reaction is one-half order with respect to the catalytic product in the case of CLA, while it is first order in the case of linoleic acid [4].

Thus, the total replacement of sunflower oil by high CLA oil for industrial frying is limited by its high polyunsaturated lipid profile specially the conjugated unsaturated fatty acid content. A reduction of 30% of the oxidative stability of oil could be registered using CLA oil for commercial frying. It could be a limiting factor to use in certain industrial food applications which involve using high temperatures (up to 140 °C), especially in presence of air. 

## 4. Conclusions

This work provides an acceptable and practical model to predict, on a laboratory scale, the industrial oxidative behavior of oil with high conjugated linoleic acid content. As expected, temperature was the most important factor affecting lipid oxidation of CLA oil. In particular between 80 °C and 150 °C, since stability of CLA show a tendency to stay constant at temperature values higher 170 °C. Although the air flow contribution was statistically significant, its interaction with the temperature contributed in a large extent to the model. It is deduced that compared to sunflower oil, CLA’s greater sensitivity to oxidation is a limiting factor for frying processes that employ high temperatures (<140 °C). Thus, for industrial frying proposes the total replacement of sunflower oil by the conjugated functional oil would increase (31%) oxidation rate. Thus, it is recommended to use high CLA oil in food industrial processes conducted at lower temperatures. Consequently, thanks to the mathematical model, a good choice of the appropriate industrial food process can be selected to avoid the oxidation of the bioactive isomers, ensuring its functionality in novel applications.
